# Machine Learning Analysis of Post-Operative Tumour Progression in Non-Functioning Pituitary Neuroendocrine Tumours: A Pilot Study

**DOI:** 10.3390/cancers16061199

**Published:** 2024-03-19

**Authors:** Ziad Hussein, Robert W. Slack, Stephanie E. Baldeweg, Evangelos B. Mazomenos, Hani J. Marcus

**Affiliations:** 1 Department of Diabetes & Endocrinology, Sheffield Teaching Hospitals NHS Foundation Trust, Sheffield S10 2JF, UK; 2 Department of Diabetes & Endocrinology, University College London Hospital NHS Foundation Trust, London NW1 2BU, UK; 3 Centre for Obesity & Metabolism, Department of Experimental & Translational Medicine, Division of Medicine, University College London, London WC1N 3BG, UK; 4 Wellcome/EPSRC Centre for Interventional and Surgical Sciences, University College London, London WC1E 6BT, UK; robert.slack.18@ucl.ac.uk; 5 Department of Medical Physics and Biomedical Engineering, University College London, London WC1E 6BT, UK; 6 Department of Neurosurgery, National Hospital for Neurology and Neurosurgery, London WC1N 3BG, UK; h.marcus@ucl.ac.uk

**Keywords:** non-functioning pituitary neuroendocrine tumours, macroadenoma, progression, recurrence, machine learning, logistic regression, knn, svm, decision tree

## Abstract

**Simple Summary:**

Effective models for predicting non-functioning pituitary neuro-endocrine tumours (NF PitNET) recurrence and regrowth following surgical intervention remain elusive. Previous studies have identified conflicting risk factors in predictions of tumour progression for patients receiving surgery for NF PitNET. The aim of this study was to develop machine learning (ML) models to improve prediction of post-operative NF PitNET progression up to 15 years following surgery. ML models were shown to be effective for predicting tumour remission, stability, and regrowth, but were non-performant when predicting tumour recurrence or reduction in size. The extent of surgical resection was shown to have the strongest influence in the performant models, with lesser influence from age, tumour volume, and the use of post-operative radiotherapy, and with no influence shown from pre- or post-operative endocrine function.

**Abstract:**

Post-operative tumour progression in patients with non-functioning pituitary neuroendocrine tumours is variable. The aim of this study was to use machine learning (ML) models to improve the prediction of post-operative outcomes in patients with NF PitNET. We studied data from 383 patients who underwent surgery with or without radiotherapy, with a follow-up period between 6 months and 15 years. ML models, including k-nearest neighbour (KNN), support vector machine (SVM), and decision tree, showed superior performance in predicting tumour progression when compared with parametric statistical modelling using logistic regression, with SVM achieving the highest performance. The strongest predictor of tumour progression was the extent of surgical resection, with patient age, tumour volume, and the use of radiotherapy also showing influence. No features showed an association with tumour recurrence following a complete resection. In conclusion, this study demonstrates the potential of ML models in predicting post-operative outcomes for patients with NF PitNET. Future work should look to include additional, more granular, multicentre data, including incorporating imaging and operative video data.

## 1. Introduction

Pituitary neuroendocrine tumours are benign adenomas of the adenohypophysis, but may exhibit aggressive and invasive behaviour [1]. Non-functioning pituitary neuroendocrine tumours often present late with invasion and compression of critical structures in the immediate vicinity of the sellar region [2,3,4]. Surgical excision via transsphenoidal route is the gold standard approach to alleviate mass effect and preserve the optic pathway. Advancements in surgical techniques over recent years may allow for a more complete tumour resection, which is crucial to reducing the risk of tumour recurrence [4,5,6]. Adjuvant radiotherapy is another treatment modality that can be utilised when surgery is not feasible or to control progressive residual disease [7,8].

The consensus for patients with NF PitNET is to undergo long-term postoperative surveillance with pituitary imaging for early detection of tumour recurrence and regrowth, which remain challenging to predict and can be associated with many tumour-related comorbidities. However, the timing and frequency of imaging vary greatly in clinical practice. Currently, ascertaining the likelihood of recurrence/regrowth of NF PitNET poses a considerable challenge. The utilisation of markers such as Ki-67, P53, or transcription factors is contingent upon the examination of tumour pathology following resection. Conversely, the reported factors available for preoperative prediction are limited [9,10,11,12,13].

Machine learning algorithms have been shown to have the ability to identify tumour risk factors and surgical outcomes [14,15,16,17]. Prediction models have the potential to be built for the purpose of identifying treatment response and predicting the rate of tumour recurrence in patients with PitNET. This might potentially lead to improved management strategies for these patients.

In this study, the authors sought to build a model using supervised machine learning to predict surgical outcome of NF PitNET following surgery with and without radiotherapy.

## 2. Methods

The study received ethics approval from the Westminster Research Ethics Committee on 7 April 2020. The publication was prepared using the Transparent Reporting of a Multivariable Prediction Model for Individual Prognosis Or Diagnosis (TRIPOD) Statement [18]. The present investigation constituted a retrospective cohort study conducted at a single medical centre. It encompassed all individuals who underwent surgical excision for NF PitNET between the time frame of 1987 and 2018. Furthermore, the study required a minimum follow-up period of six months for inclusion. The research was carried out at the National Hospital for Neurology and Neurosurgery, which is affiliated with University College London Hospitals in London. Surgery was primarily conducted by three highly skilled neurosurgeons. A retrospective analysis of medical case records was conducted. The diagnosis of NF PitNET was established by considering the lack of clinical and biochemical indications of active tumours. Information regarding the demographic characteristics of patients, the various treatment methods employed, as well as the occurrence and regrowth of tumours, was gathered and documented. The complete compilation of fields and their corresponding descriptions can be located in Appendix A.

**Figure 1:**
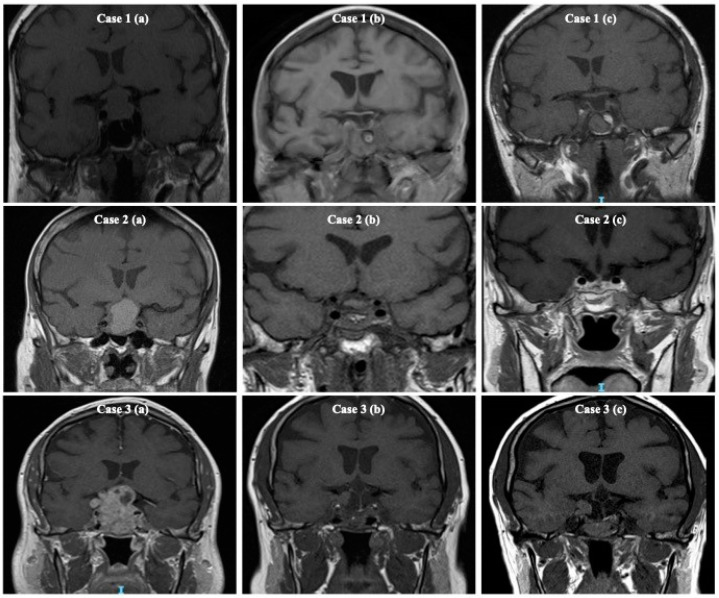
T1-weighted coronal Magnetic Resonance Imaging (MRI) scans demonstrating the degree of surgical excision in patients with non-functioning pituitary neuroendocrine tumours. The appearance of imaging is classified into three categories: complete resection, residual tumour, and sellar tissue of uncertain significance. These classifications are based on the visual appearance of the imaging results after the surgical procedure. In Case 1, the imaging protocol consisted of three distinct time points: (**a**) a preoperative MRI scan, (**b**) the first postoperative MRI scan indicating full excision, and (**c**) a follow-up MRI scan performed 5 years after the surgical intervention. In Case 2, the patient underwent a preoperative magnetic resonance imaging (MRI) scan (**a**). Subsequently, a first post-operative MRI was conducted (**b**) which revealed the presence of residual sellar tissue with undetermined relevance, as stated. Finally, a follow-up period of 5 years was observed after the surgery (**c**). In Case 3, (**a**) MRI scan before surgery, (**b**) the first postoperative MRI revealing evident residual disease, and (**c**) the 5-year follow-up MRI conducted after surgery. The image has been utilised in accordance with the permissions granted by Frontiers in Surgery [9].

The decision of surgical excision for each patient was reached subsequent to careful discussion in the pituitary multidisciplinary meeting at our institution. Patients who had NF Pit NET causing compression of the optic pathway or were adenomas that were growing and posing a threat to the optic chiasm underwent primary surgery. Immunohistological examination was performed on all tumour tissues to ascertain the specific type of pituitary adenoma. The MIB-1 monoclonal antibody was employed for the purpose of identifying the presence of the Ki-67 antigen in tissues that had been fixed in formalin and embedded in paraffin. A high level of Ki-67 expression was defined as above 3% according to previous studies [19,20]. The degree of surgical resection was assessed in all patients by post-operative imaging techniques, either Magnetic Resonance Imaging (MRI) or, in cases where MRI was not feasible, Computed Tomography (CT). These imaging procedures were conducted between 3–6 months after the surgery. The neuroradiologists independently described radiological imaging as either full resection, incomplete resection, or residual tissue of uncertain significance (Figure 1) [9]. Tumour recurrence was defined as the reappearance of a tumour on subsequent radiological imaging after complete surgical removal. On the other hand, regrowth was characterised by the emergence of residual tumour tissue following an incomplete surgical resection, as assessed by an independent neuroradiologist. The decision to perform a surgical procedure and administer radiotherapy was determined by a comprehensive evaluation of the patient’s visual fields, a review of relevant imaging results, deliberation in a multidisciplinary meeting, and consideration of patient’s personal preferences.

The data were first gathered using Microsoft Excel v2307 and then imported into a set of specialised Python v3.9.7 (64-bit|Windows 10) scripts for the purpose of analysis and visualisation. Reference to the specific software environment can be found in Appendix A, Table A1. Automated procedures were implemented to facilitate the conversion of text, numbers, and dates. Specifically, for date fields, the system verified the necessary fields against a range of potential acceptable forms and converted them to an ISO standard data type if feasible.

In anticipation of the use of machine learning algorithms, more advanced timetables were developed for individual patients in relation to radiological results. The inclusion of these timeframes was crucial to providing a comprehensive sequence of radiological observations that offer sufficient detail to gain clinical understanding and ensure consistency for analysis throughout the whole patient cohort. Radiological outcomes were determined based on the closest radiological assessment to the start-date of each period. For ease of reporting, initial post-operative scans showing complete resection were categorised as “No Residual Tumour”, and all other residual tumour classifications were categorised as “Residual Tumour Stable” until such time as an increase or reduction in size was exhibited. Incorporation of resection and residual tumour data into both the radiology timeline and the features being used to make predictions led to overstated accuracy when predicting early tumour progression in patients yet to receive another post-operative scan. This was considered appropriate, given the importance of resection completeness as a predictive feature of tumour recurrence and regrowth in previous studies, and given that any increase in predictive performance would be experienced equivalently across ML algorithms and with the logistic regression model being used as a comparative. The same parameters (radiological, endocrinological outcomes, and demographics) used to develop the ML models were used for logistic regression. This allowed a fair comparison between logistic regression and the proposed ML models.

The investigation was conducted by filtering the radiological result timings to allow the focused application of machine learning methods. Machine learning techniques were used for each successive period of radiological scans, with a maximum duration of 15 years. To ensure optimal clinical applicability, the radiological outcomes were refined by excluding secondary outcomes such as optic nerve contact/compression and cavernous invasion. The endocrine profiles were aligned with the temporal periods of radiological outcomes, including a pre-operative profile. Subsequently, additional features included in the source data were given the opportunity to be incorporated into the machine learning dataset, with the consideration of suitable normalisation techniques for factors such as age and tumour volume. Features that include just two potential values, such as sex, were simplified into a single Boolean characteristic. Features that provide various outputs, such as the Second Radiology Scan, were transformed into two distinct features to account for each potential outcome, as shown by the Second Scan. Complete Resection: True, Second Scan Complete Resection: False. This approach mitigated the possible influence of unrecorded outcomes on the machine learning models, enabling the algorithms to consider both the presence and absence of a certain outcome when identifying the optimal predictive model.

Machine learning classification techniques were used in order to forecast the subsequent behaviour of tumours after the first surgical procedure over a period of up to 15 years. The algorithms that were evaluated in this study were basic logistic regression, k-nearest neighbour (KNN), support vector machine (SVM), and decision tree. The selection of these methods was based on their superior performance in classifying models when applied to datasets of comparable size [21]. Given the limited size of the sample, the training data was generated by randomly selecting 80% of the patients. Subsequently, the test dataset, comprising the remaining 20%, was used for model evaluation. The performance of the models was evaluated based on metrics such as accuracy, F1 score, precision, recall, and the Area Under the Receiver Operating Characteristic curve (AUC-ROC). A confusion matrix was produced for each scenario. The One-vs-Rest (OvR) approach was used for model development and metric computation in the context of a multiclass challenge.

In order to address the possibility of model instability, the algorithm under test was executed 20 times, and the resultant output was converted to a mean and standard deviation for each metric. The algorithms’ performance was evaluated by comparing their prediction power for radiological outcomes at certain time points after the surgical procedure, including 6 months, 1 year, 5 years, and 10 years. The decision tree technique was used to gather data pertaining to feature significance, thereby enabling the identification of the most significant characteristics for each outcome period.

Following model execution across all tumour progression outcomes, the SVM and decision tree models were rerun with the outcome under prediction limited to recurrence or regrowth at and beyond 2 years post-operatively (earlier post-operative periods showed too few patients in these classes to be effectively modelled).

## 3. Results

### 3.1. Population Statistics

Simple population statistics for the patient cohort are shown in Table 1. Knosp classification and size of non-functioning pituitary adenomas on imaging before surgery are demonstrated in Table 2. 

Complete surgical resection was achieved in 229 patients (60%).

### 3.2. Cumulative Probability of Recurrence and Regrowth

**Figure 2:**
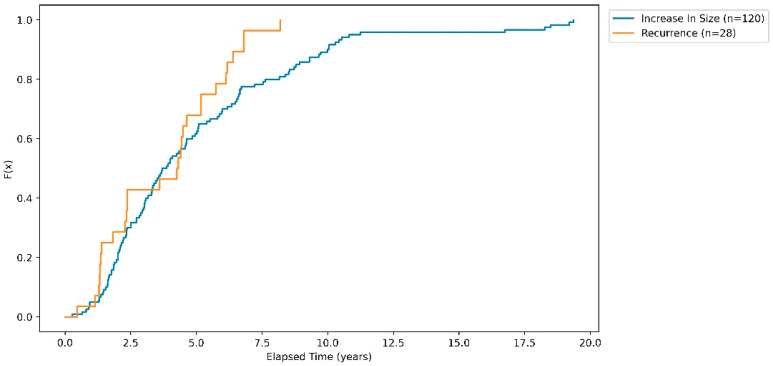
Kaplan–Meier curve showing probability as a function of time for populations exhibiting post-operative tumour recurrence and regrowth.

Figure 2 features a Kaplan–Meier curve, showing the cumulative risk over time of post-operative tumour recurrence and regrowth for the populations exhibiting these outcomes.

### 3.3. Derived Radiological Outcome Timelines

**Figure 3:**
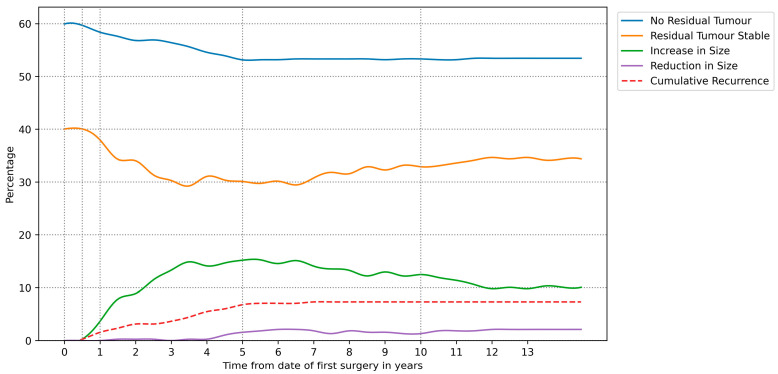
Radiological outcome populations as a function of time following the first surgery in patients with non-functioning pituitary adenomas.

Figure 3 shows the radiological outcome rates as a function of time for the derived timelines, with specific rates shown at time of surgery and 6 months, 1 years, 5 years, and 10 years post-operatively, selected for clinical significance, included as Table A2 in Appendix A. 

### 3.4. Machine Learning for Determining Post-Operative Radiological Outcome

#### 3.4.1. Model Performance Analysis

Model accuracy and AUC-ROC comparison between the selected algorithms is shown in Table 3 as a mean and standard deviation across all prediction periods and classes.

#### 3.4.2. Feature Importance in Determining Radiology Outcomes

Table 4 shows the derived feature importance as mean ± standard deviation for each decision tree model, excluding features with a mean importance of less than 0.2, or where the standard deviation of the importance was greater than or equal to the mean. Boolean features where both positive and negative results contribute strongly are shown regardless of standard deviation, as this value will appear inflated due to the sensitivity of the model to Boolean decisions.

#### 3.4.3. Predicting Tumour Recurrence and Regrowth

A breakdown of the AUC-ROC scores for each radiology outcomes is shown in Table 5. No patients exhibited a reduction in size within a year of initial surgery.

The SVM and decision tree models were subsequently executed limiting each to predicting a single Boolean outcome, either for tumour recurrence or regrowth. Due to the limited number of early post-operative results in either class, both models began predictions at 2 years post-operative. The AUC-ROC scores for each model are shown in Table 6.

## 4. Discussion

Machine learning models possess the ability to acquire the most optimal characteristics for predicting outcomes in clinical practice. Instead of relying on a human operator to manually identify these data, which is time-consuming and requires a lot of effort, machine learning models may automatically discover the most reliable predictive variables and potentially apply this knowledge to improve patients’ outcomes. Our work serves as a pilot study to elucidate the applicability of ML models, based on a number of peri-operative parameters, typically recorded in PitNET, for predicting post-operative tumour progression.

Limited models exist for predicting post-operative outcomes for patients receiving surgery for NF PitNET. The existing literature provides conflicting evidence of factors affecting tumour progression, although complete resection is indicated in many studies as reducing the risk of recurrence and regrowth. ML models have shown strong predictive capabilities for tumour progression, but the most performant of these use neuroimaging data as the basis for prediction. This study applied ML algorithms in predicting post-operative tumour progression for a large cohort of patients with NF PitNET, with data covering presentation, details of interventions, tumour characteristics, Ki-67, and follow-up results of radiology scans. SVM and decision tree models were successful in outperforming conventional statistical models in predicting radiological outcomes for patients with NF PitNET. SVM was shown as the strongest ML model for predicting tumour progression (mean accuracy 0.82; mean AUC-ROC 0.67) when compared with logistic regression (mean accuracy 0.67; mean AUC-ROC 0.64). This model showed a strong association between tumour progression and resection completeness, with weaker associations with age, tumour size, and the use of postoperative radiotherapy. Our results indicate that the relationships between these parameters and post-operative outcomes are better described using non-linear modelling (i.e., SVM) than a typical statistical approach (logistic regression). 

Surgical resection via transsphenoidal route remains the gold standard for treating NF PitNET with pressure effect on the nearby crucial structures. Tumour recurrence remains a risk, even in cases of complete resection, with no consistent link shown between risk factors and tumour recurrence/regrowth. Post-operative tumour growth for patients with residual tumour is generally slow, but follow-up surgeries and/or radiotherapy are indicated in cases where regrowth is aggressive, or in the presence of mass-effects.

Several studies have shown an inverse correlation between radiologically assessed complete resection of NF PitNET and tumour recurrence and regrowth [22,23,24], however, this was not supported by a meta-analysis conducted by Roelfsema et al. in cases of recurrence following remission [25]. PitNET invasion of the parasellar spaces is considered one of the limiting factors to achieving complete resection posing the subsequent risk of tumour progression following operation. Magnetic resonance imaging (MRI) is the main diagnostic technique used to assess tumour extension. The utilisation of high-field strength 3 Tesla scanners has become prevalent in clinical settings, resulting in substantial enhancements in image quality and spatial resolution and being superior to conventional 1.5T MRI in accurately delineating the extent of surgical resection and the presence of residual disease [25,26]. Prospective research is required to explore the use of such scanners with ML to assess the prediction of surgical resection degree in patients with PitNET. Within this cohort of 383 patients, 229 (60%) exhibited total surgical resection as an outcome of their first surgery, with recurrence following complete surgical removal identified in 26 (7%) within 5 years of initial surgery, and 28 (7%) within 10 years of initial surgery. The number of patients exhibiting tumour regrowth after incomplete resection rose in the years following surgery, reaching a maximum of 58 patients (15%) at 5 years post-operatively. 

**Figure 4:**
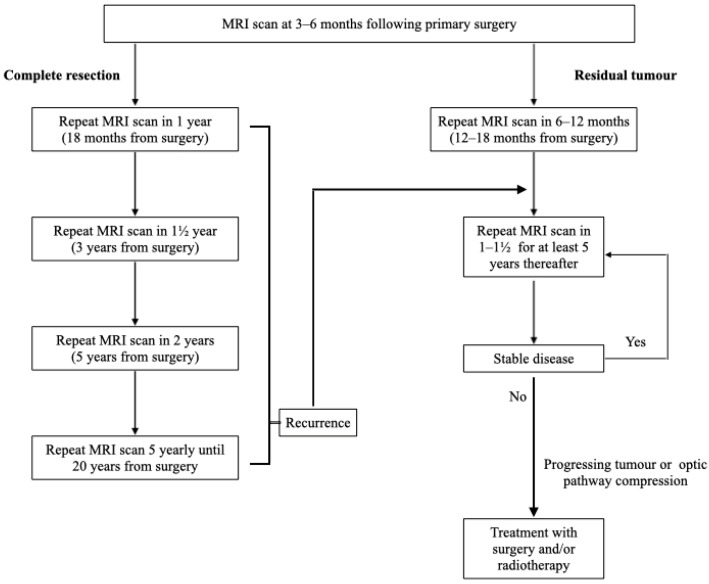
Imaging surveillance plan for patients with non-functioning pituitary neuroendocrine tumours following primary surgery. MRI: Magnetic Resonance Imaging. permission granted by Frontiers in Surgery [9].

Our study has shown superior accuracy and AUC-ROC performance of SVM (accuracy: 0.82; AUC-ROC: 0.67) and decision tree (accuracy: 0.78; AUC-ROC: 0.66) models over conventional logistic regression (accuracy: 0.67; AUC-ROC: 0.64) when predicting future radiology outcomes. In the case of the decision tree model, the highest influence in predicting post-operative tumour progression came from the extent of surgical resection, supporting studies showing a similar correlation. Crucially, however, analysis of AUC-ROC scores for individual outcomes shows that while these models are strong predictors of continued remission, stable tumours, and tumour regrowth (mean ± std AUC-ROC of 0.91 ± 0.03, 0.78 ± 0.06 and 0.68 ± 0.1, respectively, at 5 years post-operative, the latter rising to 0.79 ± 0.07 when modelled in isolation), they perform no better than chance in their predictions of recurrence or tumour reduction. This may be in part due to the small number of patients exhibiting these radiology results at a point in time, with maximum population percentages of 1% and 2% across the “Recurrence” and “Reduction in Size” groups, respectively, in any one prediction period. However, when limiting the models to predict recurrence in isolation from other outcomes, both the SVM and decision tree models showed consistent AUC-ROC scores in the range 0.49–0.5 across all prediction periods, supporting the existing literature in showing no strong indicator within this dataset for predicting post-operative recurrence of NF PitNET following complete resection. Therefore, long term imaging surveillance is warranted even following complete resection, albeit at a reduced frequency compared to patients with residual disease. The authors of this study have proposed a long-term follow-up strategy for patients with NF PitNET following primary surgery, shown in Figure 4 [9].

## 5. Conclusions

ML models have been shown to deliver strong predictive performance when compared with conventional statistical methods for predicting post-operative tumour progression following surgical intervention. Models were executed over a feature-rich, single-centre dataset of 383 patients, which included details of population demographics, endocrine profiles, treatment modalities and results, and assessments of pre and post-operative radiology scans. SVM and decision tree models showed strong performance in the prediction of remission, stability, and regrowth, with predictions strongly influenced by the extent of surgical resection, and with lesser influence from age, tumour volume, and the use of post-operative radiotherapy. SVM showed the strongest overall performance (accuracy: 0.82; AUC-ROC: 0.67) when compared with conventional logistic regression (accuracy: 0.67; AUC-ROC: 0.64). No models successfully predicted recurrence or tumour reduction, suggesting a lack of association between these outcomes and the patient and treatment features included in this study. No association was shown between pre- and post-operative endocrine function and post-operative tumour progression. ML models can be employed to predict tumour progression and endocrine outcomes in NF PitNET, as demonstrated in this study. Further research is required to further develop and optimise high-performing ML models to aid in identifying post-operative risk factors for patients treated for NF PitNET. Patients exhibiting residual tumour are at a higher risk of post-operative regrowth, and these patients require long-term monitoring using neuroimaging to ensure timely interventions and optimise patient outcomes.

## Figures and Tables

**Table 1:** Patient cohort summary.

Measure	Value
Total number of patients	383
Average age	56.8
Standard age (in years)	13.5
Sex ratio, expressed as the ratio of males to females.	67:33
Percentage of patients underwent one or more surgeries	100
Percentage of patients who had two or more surgeries	22
Percentage of patients who had three surgeries	4
Percentage of patients had radiotherapy	17
Mean follow-up (range in years)	8 (0.5–23)
Total number of patients who had follow up between 0–5 years	86
Total number of patients who had follow up between 5–10 years	195
Total number of patients who had follow up between 10–15 years	92

**Table 2:** Knosp classification (expressed in percentage), and dimensions and volume of non-functioning pituitary adenomas on preoperative imaging with the risk of recurrence and regrowth following primary surgery. Cm: Centimetre; IQR: Interquartile Range. permission granted by Frontiers in Surgery [9].

Knosp Classification on Preoperative Imaging of Non-Functioning Pituitary Macroadenomas			
Grade I		38%	
Grade II		33%	
Grade III A		17%	
Grade III B		2%	
Grade IV		11%	
Dimensions and Volume on Preoperative Imaging			
	Tumour recurrence/regrowth	No tumour recurrence/regrowth	*p* value
Craniocaudal diameter (IQR)	3.2 cm (2.4–3.9)	2.5 cm (1.9–3.2)	0.001
Transverse diameter (IQR)	2.7 cm (2.1–3.0)	2.2 cm (1.8–2.7)	0.001
Anteroposterior diameter (IQR)	2.3 cm (1.9–2.6)	1.9 cm (1.6–2.3)	0.001
Volume (IQR)	10.5 cm^3^ (5–16)	5.8 cm^3^ (3–10)	0.001

**Table 3:** Comparison of tumour progression model performance, taken as a mean and standard deviation of accuracy and AUC-ROC for all future periods being modelled.

	Accuracy		AUC-ROC	
	Mean	Std	Mean	Std
Logistic Regression	0.67	0.06	0.64	0.08
KNN	0.74	0.05	0.62	0.03
SVM	0.82	0.04	0.67	0.02
Decision Tree	0.78	0.05	0.66	0.05

**Table 4:** Mean ± Standard Deviation feature importance in predicting radiological outcomes following first surgery.

Feature	Feature Importance (Mean ± Std) at Post-Operative Prediction Period			
	6 Months	1-Year	5 Years	10 Years
Second Scan Complete Resection: True	0.33 ± 0.34	0.31 ± 0.32	0.32 ± 0.27	0.33 ± 0.28
Second Scan Complete Resection: False	0.33 ± 0.34	0.31 ± 0.32	0.21 ± 0.27	0.22 ± 0.28
Age	0.10 ± 0.03	0.11 ± 0.03	0.11 ± 0.03	0.11 ± 0.04
Tumour Volume (cc)	0.08 ± 0.04	0.09 ± 0.04	0.09 ± 0.03	0.09 ± 0.03
Radiotherapy Administered: True			0.03 ± 0.02	0.03 ± 0.02

**Table 5:** Mean ± Standard Deviation Area Under Curve—Receiver Operating Curve (AUC-ROC) scores for each radiology outcome, as predicted by decision tree model using pre-operative endocrine profiles.

Radiology Outcome	AUC-ROC (Mean ± Std) at Post-Operative Prediction Period			
	6 Months	1-Year	5 Years	10 Years
No Residual Tumour	0.99 ± 0.02	0.98 ± 0.02	0.91 ± 0.03	0.88 ± 0.05
Residual Tumour Stable	1 ± 0.01	0.92 ± 0.06	0.78 ± 0.06	0.78 ± 0.08
Increase in Size	0.5 ± 0	0.52 ± 0.07	0.68 ± 0.1	0.6 ± 0.13
Recurrence	0.5 ± 0	0.5 ± 0	0.51 ± 0.06	0.5 ± 0.01
Reduction in Size			0.5 ± 0	0.5 ± 0.01

**Table 6:** Mean ± Standard Deviation Area Under Curve—Receiver Operating Curve (AUC-ROC) scores for SVM and decision tree models predicting tumour recurrence and regrowth in isolation at 2, 5, and 10 years post-operatively.

Radiology Outcome	ML Model	AUC-ROC (Mean ± Std) at Post-Operative Prediction Period		
		2 Years	5 Years	10 Years
Increase in Size	SVM	0.5 ± 0	0.86 ± 0.09	0.88 ± 0.04
	Decision Tree	0.63 ± 0.13	0.79 ± 0.07	0.85 ± 0.07
Recurrence	SVM	0.5 ± 0	0.5 ± 0	0.5 ± 0
	Decision Tree	0.5 ± 0.01	0.49 ± 0.01	0.49 ± 0.03

## Data Availability

The data presented in this study will be made publicly available, upon the paper’s acceptance, in the University College London institutional research repository (https://rdr.ucl.ac.uk/).

## Appendix A

**Table A1:** Software packages used for algorithm development.

Software/Library	Version
Spyder IDE	5.1.5
Python	3.9.7 (64-bit)
Qt	5.9.7
PyQt	5.9.2
MS Windows	10
Pandas	1.3.4
Sklearn	1.1.2
Matplotlib	3.4.3
Seaborn	0.11.2

**Table A2:** Population percentages for radiological scan outcomes over clinically relevant periods, extracted from derived patient timelines.

	Time Relative to First Surgery				
	0–6 Months	6–12 Months	1–1.5 Years	5 Years	10 Years
No Residual Tumour	59.9%	59.7%	58.4%	53.1%	53.3%
Residual Tumour Stable	40.1%	39.8%	36.4%	29.3%	32.9%
Increase in Size	0.0%	0.3%	3.7%	15.2%	12.5%
Reduction in Size	0.0%	0.0%	0.0%	1.6%	1.3%
Cumulative Recurrence	0.0%	0.3%	1.6%	6.8%	7.3%
